# Associations of diarrhea episodes and seeking medical treatment among children under five years: Insights from the Zimbabwe Demographic Health Survey (2015–2016)

**DOI:** 10.1002/fsn3.2596

**Published:** 2021-09-21

**Authors:** Godfrey Musuka, Tafadzwa Dzinamarira, Grant Murewanhema, Diego Cuadros, Innocent Chingombe, Helena Herrera, Felicia Takavarasha, Munyaradzi Mapingure

**Affiliations:** ^1^ ICAP at Columbia University Harare Zimbabwe; ^2^ School of Health Systems & Public Health University of Pretoria Pretoria South Africa; ^3^ Unit of Obstetrics and Gynaecology Faculty of Medicine and Health Sciences University of Zimbabwe Harare Zimbabwe; ^4^ Department of Geography and Geographic Information Science University of Cincinnati USA; ^5^ University of Portsmouth Portsmouth UK; ^6^ Independent Consultant Amsterdam Netherlands

**Keywords:** diarrhea, medical treatment, under 5, Zimbabwe Demographic and Health Survey

## Abstract

Diarrhea is a significant pediatric public health concern globally and places a significant burden on healthcare systems. In resource‐limited settings, the problems of diarrhea could be worse than reported. Continuously monitoring and understanding the changing epidemiology of diarrhea, including risk factors, remain an important aspect necessary to design effective public health interventions to reduce the incidence, outcomes and strain on healthcare resources caused by diarrheal illness. We, therefore, undertook this study to understand the factors associated with diarrhea as well as describe determinants for seeking medical treatment in children under‐five in Zimbabwe using the Zimbabwe Demographic and Health Survey 2015–2016 Data. Children with recent diarrhea were on average younger (mean age 22 months), compared to those who did not have an episode of diarrhea (mean age 30 months) *p* = .001. Incidence of recent diarrhea was lower among female children compared to their male counterparts (16% vs. 19%), *p* = .013. Incidence of diarrhea decreased with increasing maternal education level and so was the same for increasing wealth quintile. Those with unimproved sources of drinking water had a higher incidence of diarrhea. The wealth quintile remained the only factor associated with seeking medical attention for a recent diarrhea episode among children less than 6 years, with those in the highest wealth quintile being 2.49 times likely to do so, *p* = .031. The results are useful in informing pediatric public health policies and strategies for them to be successful in significantly reducing the incidence, morbidity, mortality and significant healthcare costs and burden to society associated with caring for children with diarrheal illnesses.

## Graphical abstract

Diarrhea is a significant paediatric public health concern globally and places a significant burden on healthcare systems. Continuously monitoring and understanding the changing epidemiology of diarrhea, including risk factors, remains an important aspect necessary to design effective public health interventions to reduce the incidence, outcomes and strain on healthcare resources caused by diarrheal illness. We, therefore, undertook this study to understand the factors associated with diarrhea as well as describe determinants for seeking medical treatment in children under five in Zimbabwe using the Zimbabwe Demographic and Health Survey 2015–2016 Data.

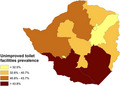

## 1 INTRODUCTION

Diarrhea remains a significant pediatric public health challenge globally (Ugboko et al., 2020). Diarrheal illnesses feature consistently in the top five causes of under‐five morbidity and mortality and place a significant burden on healthcare systems (Mokomane et al., 2018; Ugboko et al., 2020). Globally, an estimated 1.7 billion diarrhea episodes are recorded annually, with an estimated 500,000 children under five years dying of diarrhea each year (WHO, 2020). In resource‐challenged settings, the figures reported could be underestimates of the significance of the problem as a result of barriers to monitoring and reporting. Integrated Disease Surveillance and Response mechanisms in place for epidemic‐prone diseases may not be as efficient in such areas. Diarrhea episodes contribute significantly to general hospital admissions particularly in the under 5 years old age group. These can cause or contribute significantly to nutritional disorders, worsen underlying medical conditions and can be worsened by underlying comorbidities (Webb & Cabada, 2018).

The etiology of diarrhea in children is multifactorial, involving contributing pathogens and predisposing conditions (Mokomane et al., 2018). Rotavirus is the most important pathogen globally, and in sub‐Saharan Africa (SSA), almost 40% of deaths caused by diarrhea are attributable to this virus in children under 5 (Troeger et al., 2018). While *Escherichia coli* is the second most important pathogen, others, such as *Salmonella* spp., *Shigella* spp., *Entamoeba histolytica*, *Bacteroides fragilis* and *Campylobacter jejuni* contribute significantly, while *Cryptosporidium* spp. may also play an important role, especially in immunocompromised children (WHO, 2017). Risk factors for diarrhea in the pediatric population include poor hygiene practices, lack of access to safe and clean water, poor nutritional status and poor sanitation (Workie et al., 2019). These risk factors are more prevalent in low‐income countries such as those in SSA.

Zimbabwe, like in other SSA settings, is resource‐challenged. Despite several years of policy and practice aiming to reduce the incidence, morbidity and mortality associated with diarrhea in the country, it remains a significant contributor to under‐five morbidity and mortality (Mukaratirwa et al., 2018). Understanding the changing epidemiology, including risk factors, remains an important aspect necessary to design effective public health interventions to reduce the incidence, outcomes and strain on healthcare resources caused by diarrheal illness. A previous study from Zimbabwe reported that direct medical costs were the primary determinant of diarrhea illness costs (Mujuru et al., 2020). Additionally, it reported that, the median total cost of a diarrhea illness resulting in hospitalization was $293.74 (IQR: 188.42, 427.89). Direct medical costs, with a median of $251.74 (IQR: 155.42, 390.96), comprised the majority of the total cost. Among children who tested positive for rotavirus, the median total illness cost was $243.78 (IQR: 160.92, 323.84). The median direct medical costs were higher for malnourished than well‐nourished children (*p* < .001). Another study reported that household flooring is an important pathway for the transmission of diarrheal pathogens, even in settings where other aspects of sanitation are sub‐optimal (Koyuncu et al., 2020). Improvements to household flooring do not require behavior change and may be an effective and expeditious strategy for reducing childhood diarrheal illness irrespective of household access to improved water and sanitation. A recent study from Zimbabwe also reported on the impact of behavior change to promote hand washing with soap (Inauen et al., 2020). The analyses of the mechanisms revealed important insights into the active ingredients of the intervention, which will facilitate its future refinement. Because of the dynamic nature of the known risk factors, it remains important to continuously monitor these parameters to effectively inform public health policy. We, therefore, undertook this study to understand the factors associated with diarrhea in children under‐five in Zimbabwe.

## 2 MATERIALS AND METHODS

### 2.1 Study area and data sources

The study area was Zimbabwe, a landlocked country in southern Africa. The Zimbabwe Demographic and Health Survey (ZDHS) methodology has been described elsewhere (ICF International, 2016). The DHS methodology has been used to collect, analyze and disseminate accurate and representative data on population demographics and health through more than 400 surveys in over 90 countries. This survey is conducted in Zimbabwe every five years, with the latest survey data collected in the last quarter of 2015 and the first quarter of 2016. Participants were enrolled in the ZDHS via a two‐stage sampling procedure to select households. A total of 400 ZDHS sample locations were identified. The study population included 5704 mothers who had at least one child aged between 0 and 59 months. A questionnaire was administered to the participants to gather information on children's health, including episodes of diarrhea and whether medical treatment for the diarrhea was sought. The questionnaire also gathered information about the sanitary conditions the children were living in. Procedures and questionnaires for standard Demographic Health Surveys (DHS) have been reviewed and approved by the ICF International Institutional Review Board (IRB). Additionally, country‐specific DHS survey protocols are reviewed by the ICF IRB and typically by an IRB in the host country specifically the Medical Research Council of Zimbabwe (MRCZ). Written informed consent was obtained from all ZDHS participants.

### 2.2 Statistical analysis

STATA Version 16.1, Texas USA, was used to conduct statistical analysis. We used simple proportion to describe the characteristics of the women and their children included in the analysis. Statistical significance cutoff for purposes of describing the risk factors for a recent episode of diarrhea was set at *p* < .05. Odds ratio and their 95% confidence intervals were also used to establish risk factors for episodes of diarrhea. We further determined the factors associated with seeking medical attention among those who had a recent episode of diarrhea. The outcome considered for this analysis was diarrhea. This binary outcome was fitted to univariate logistic regression models for various risk factors such as demographics. A multivariate logistic regression model was also fitted in STATA since the nature of exposures and outcomes can follow a logit model.

$$\text{Logit}\;(\pi \left(X\right)=\beta 0+\beta 1X1+\ldots +\beta pXpldots+\beta pXp$$

where the probability of the binary outcome, e.g., having had diarrhea or not can be calculated simultaneously for various risk factors combined and expressed as odds ratios.

**FIGURE 1:**
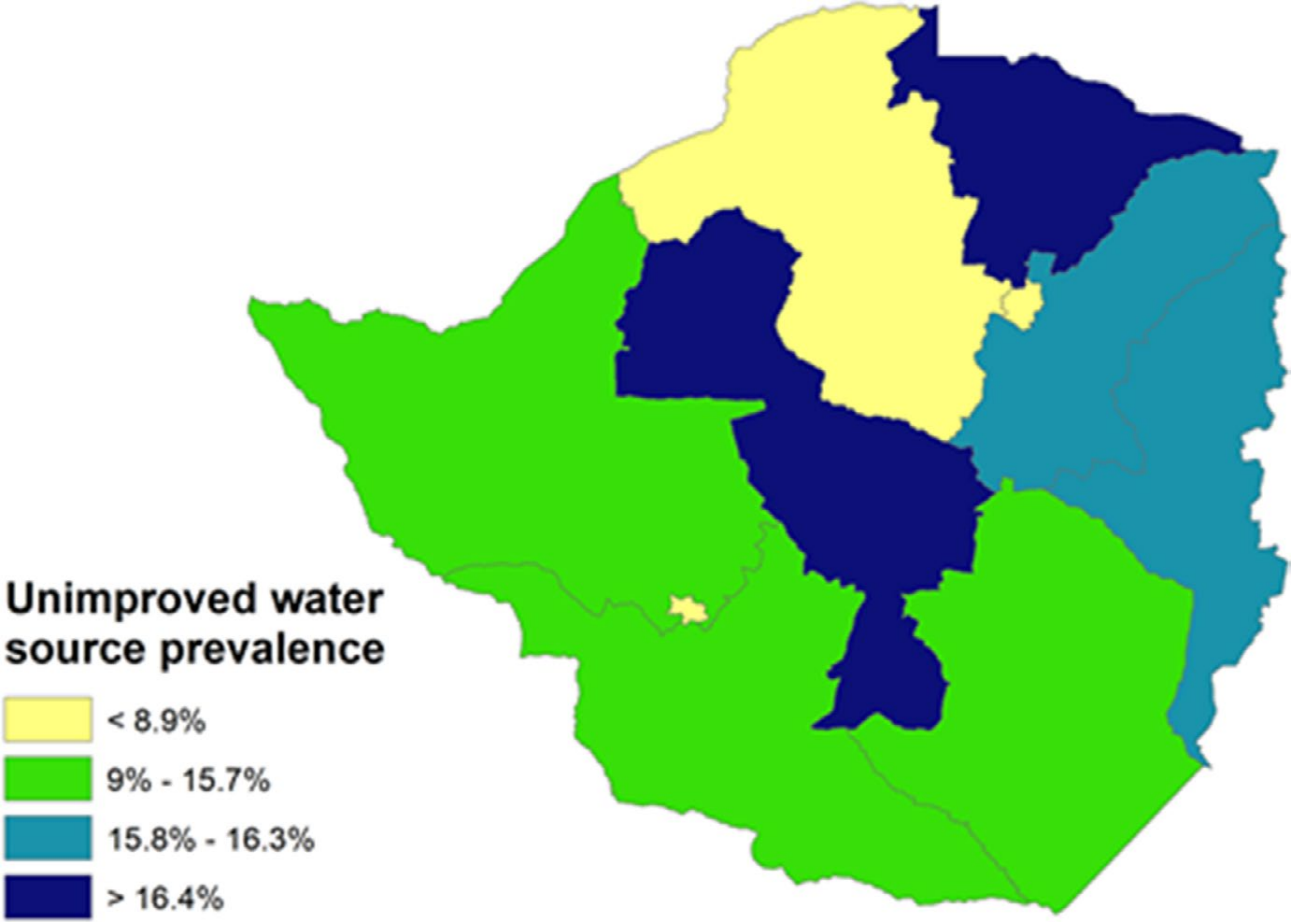
Prevalence of unimproved water source

**FIGURE 2:**
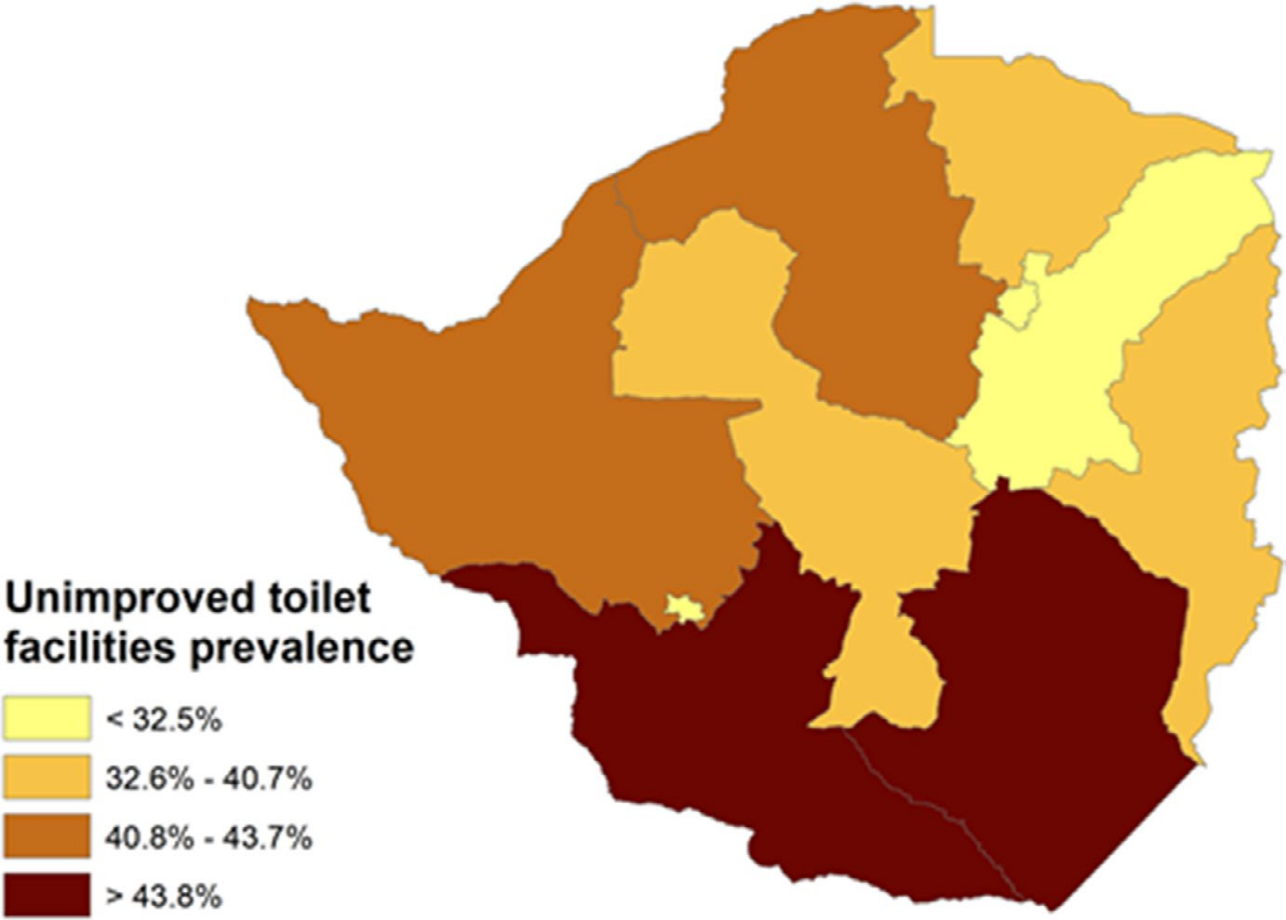
Prevalence of unimproved toilet facilities

We also generated maps to illustrate the spatial distribution of key diarrheal risk factors at provincial level. These maps were generated using ArcGIS PRO (Esri, 2020), see figure 1 and figure 2.

## 3 RESULTS

### 3.1 Demographic characteristics

From the demographics, there was an even spread of children's gender (49% males and 51% females), see Table 1. The majority (68%) of the participants lived in rural areas. Half of the participants belonged to the Apostolic Sect, and 68% of them had completed at least secondary education. Overall, 17% of the children under 5 had a recent episode of diarrhea, 19% among males and 16% among females.

**TABLE 1:** Mother and baby demographic characteristics for Zimbabwe Demographic and Health Survey 2015–2016 participants included in this analysis

Variable	Frequency *n* (%) *N* max = 5704
Baby age group in months	
<6	615 (11)
6–8	289 (5)
9–11	279 (5)
12–17	586 (11)
18–23	560 (10)
24–35	1131 (20)
36–47	1126 (20)
48–59	1118 (20)
Mother age group in years	
15–19	368 (6)
20–24	1392 (23)
25–29	1527 (27)
30–34	1317 (24)
35–39	722 (13)
40–44	323 (6)
45–49	55 (1)
Baby gender	
Male	2796 (49)
Female	2908 (51)
Residence	
Urban	2167 (32)
Rural	3537 (68)
Mother's education level	
No education	56 (1)
Primary	1657 (31)
Secondary	3641 (62)
Tertiary or higher	350 (6)
Religion	
Traditional	30 (1)
Roman catholic	276 (5)
Protestant	736 (13)
Pentecostal	1340 (21)
Apostolic sect	2648 (50)
Other Christian	314 (4)
Muslim	15 (0)
None	337 (6)
Other	8 (0)
Wealth quintile	
Poorest	1157 (23)
Poorer	1002 (20)
Middle	873 (17)
Richer	1491 (23)
Richest	1181 (17)

### 3.2 Factors associated with a recent episode of diarrhea

From univariate analysis, children with recent diarrhea were on average younger (mean age 22 months), compared to those who did not have an episode of diarrhea (mean age 30 months) *p* = .001, see Table 2. Incidence of recent diarrhea was lower among female children compared to their male counterparts (16% vs. 19%), *p* = .013. Incidence of diarrhea did not differ by area of residence. Incidence of diarrhea decreased with increasing maternal education level and so was the same for increasing wealth quintile. There was no association between the incidence of diarrhea and religion. Those with unimproved sources of drinking water had a higher incidence of diarrhea. We however did not find an association between the incidence of diarrhea and the status of toilet facilities.

**TABLE 2:** Factors associated with a recent episode of diarrhea among children aged 0–59 months

Variable	Had recent diarrhea *n* (%)	Did not have recent diarrhea *n* (%)	Unadjusted Odds Ratio (95 CI)	*p*‐value	Unadjusted Odds Ratio (95 CI)	*p*‐value
Baby age group in months Mean (*SD*)	23 (14)	30 (18)	1 0.97 (0.97–0.98)	0.001	1 0.97 (0.97–0.98)	0.000
Gender Male Female	500 (19) 431 (16)	2,296 (81) 2,477 (84)	1 0.82 (0.69–0.96)	0.013	1 0.83 (0.70–0.98)	0.031
Residence Urban Rural	347 (17) 584 (17)	1,820 (83) 2,953 (83)	1 1.01 (0.85–1.20)	0.916	1 0.75 (0.51–1.09)	0.126
Mother's education level No education Primary Secondary Tertiary or higher	12 (24) 277 (18) 615 (17) 27 (7)	44 (76) 1,380 (82) 3,026 (83) 323 (93)	1 0.70 (0.35–1.42) 0.68 (0.34–1.36) 0.25 (0.11–0.58)	0.328 0.278 0.001	1 0.77 (0.37–1.63) 0.70 (0.33–1.48) 0.30 (0.12–0.73)	0.498 0.350 0.008
Religion Traditional Roman Catholic Protestant Pentecostal Apostolic sect Other Christian Muslim None Other	9 (20) 37 (15) 108 (15) 228 (18) 449 (18) 42 (14) 3 (13) 53 (18) 2 (23)	21 (80) 239 (85) 628 (85) 1,112 (82) 2,199 (82) 272 (86) 12 (87) 284 (82) 6 (77)	1 0.70 (0.28–1.77) 0.67 (0.28–1.60) 0.85 (0.36–2.00) 0.83 (0.36–1.92) 0.63 (0.25–1.58) 0.58 (0.10–3.25) 0.85 (0.35–2.08) 1.16 (0.17–7.84)	0.454 0.371 0.714 0.658 0.325 0.533 0.720 0.882	1 0.70 (0.28–1.76) 0.62 (0.26–1.48) 0.72 (0.31–1.71) 0.66 (0.29–1.53) 0.51 (0.20–1.29) 0.45 (0.08–2.43) 0.66 (0.27–1.63) 0.73 (0.10–5.55)	0.443 0.279 0.460 0.333 0.154 0.351 0.372 0.759
Wealth quintile Poorest Poorer Middle Richer Richest	199 (19) 165 (17) 142 (17) 261 (18) 164 (14)	958 (81) 837 (83) 731 (83) 1,230 (82) 1,017 (86)	1 0.87 (0.69–1.11) 0.88 (0.68–1.14) 0.97 (0.77–1.22) 0.74 (0.57–0.96)	0.268 0.333 0.787 0.024	1 0.91 (0.70–1.17) 0.88 (0.64–1.21) 0.93 (0.61–1.41) 0.71 (0.42–1.20)	0.450 0.421 0.722 0.202
Source of drinking water Improved Unimproved	679 (16) 206 (19)	3,636 (84) 936 (81)	1 1.21 (1.00–1.45)	0.048	1 1.22 (0.99–1.52)	0.067
Toilet facilities Improved Unimproved	553 (17) 332 (17)	2,920 (83) 1652 (83)	1 1.06 (0.89–1.25)	0.521	1 0.92 (0.72–1.17)	0.491

### 3.3 Factors associated with seeking medical attention after an episode of diarrhea

In univariate analysis, those in highest wealth quintile were more likely to seek medical attention for the recent diarrhea episode among children less than 6 years, see Table 3. In multivariate analysis, baby age and baby gender remained significant factors associated with the recent incidence of diarrhea. Although of borderline significance *p* = .067, having an unimproved source of drinking water was associated with a higher incidence of diarrhea among these children.

**TABLE 3:** Factors associated with seeking medical treatment after a recent episode of diarrhea among children aged 0–59 months

Variable	Received treatment *n* (%)	Did not receive any treatment *n* (%)	Unadjusted Odds Ratio (95 CI)	*p*‐value	Unadjusted Odds Ratio (95 CI)	*p*‐value
Baby age group in months Mean (*SD*)	23 (13)	22 (15)	1.00 (0.99–1.01)	0.557	1.00(0.99–1.01)	0.555
Gender Male Female	209 (39) 179 (40)	291 (61) 252 (60)	1 1.08 (0.80–1.45)	0.631	1 1.09 (0.81–1.48)	0.557
Residence Urban Rural	157 (41) 231 (39)	190 (59) 353 (61)	1 0.93 (0.68–1.29)	0.677	1 1.70 (0.90–3.23)	0.104
Mother's education level No education Primary Secondary Tertiary or higher	7 (52) 103 (32) 264 (42) 14 (50)	5 (48) 174 (68) 351 (58) 13 (50)	1 0.44 (0.12–1.55) 0.67 (0.19–2.34) 0.90 (0.20–4.07)	0.201 0.528 0.892	1 0.36 (0.11–1.24) 0.52 (0.15–1.77) 0.54 (0.12–2.50)	0.105 0.293 0.433
Religion Traditional Roman catholic Protestant Pentecostal Apostolic sect Other Christian Muslim None Other	5 (59) 18 (43) 46 (42) 98 (42) 179 (47) 21 (66) 1 (32) 20 (32) 0 (0)	4 (41) 19 (57) 62 (58) 130 (58) 270 (62) 21 (53) 2 (34) 33 (68) 2 (100)	1 0.54 (0.11–2.57) 0.50 (0.12–2.16) 0.50 (0.12–2.08) 0.42 (0.10–1.73) 0.61 (0.13–2.87) 1.37 (0.08–23.33) 0.33 (0.07–1.52) ‐	0.436 0.354 0.34 0.231 0.531 0.827 0.156 ‐	1 0.52 (0.10–2.71) 0.48 (0.10–2.25) 0.50 (0.11–2.27) 0.47 (0.11–2.06) 0.68 (0.13–3.47) 1.87 (0.11–32.66) 0.39 (0.08–1.95) ‐	0.438 0.355 0.372 0.314 0.643 0.667 0.253 ‐
Wealth quintile Poorest Poorer Middle Richer Richest	71 (35) 67 (40) 59 (39) 105 (38) 86 (50)	128 (65) 98 (60) 83 (61) 156 (62) 78 (50)	1 1.26 (0.80–1.97) 1.19 (0.74–1.92) 1.16 (0.75–1.78) 1.85 (1.14–3.01)	0.312 0.468 0.503 0.013	1 1.26 (0.80–1.99) 1.13 (0.69–1.84) 1.48 (0.77–2.82) 2.49 (1.09–5.68)	0.32 0.638 0.236 0.031

In multivariate analysis, wealth quintile remained the only factor associated with seeking medical attention for a recent diarrhea episode among children less than 6 years, with those in the highest wealth quintile being 2.49 times likely to do so, *p* = .031.

### 3.4 Geospatial maps of water and sanitation facilities

The maps indicated that provinces like Midlands and Mashonaland Central were the provinces with the highest percentage of households lacking an adequate source of drinking water (Figure 1).

## 4 DISCUSSION

In this analytical cross‐sectional study, we examined the factors associated with episodes of diarrhea in children under five years of age. As discussed previously, understanding these factors is a critical step for designing preventive public health actions to reduce the incidence, morbidity and mortality associated with this important cause for adverse infant outcomes. Our findings are not deviant from the wider literature and serve to corroborate that some of the factors that have been identified previously are important determinants of this problem in the population studied. The risk factors are largely related to the social determinants of health and point toward a continued need for addressing these and reducing socioeconomic inequalities in Zimbabwe.

Sixty‐eight percent of the participants, constituting the majority, lived in rural areas. This is consistent with the general population demographics where the majority live in rural areas and is an indicator of the representativeness of this study. Half of the participants belonged to the apostolic sector, which is an important characteristic preventing health‐seeking behavior and health promotion uptake (UNICEF, 2011). This population has been widely acknowledged to have poorer health‐seeking behaviors when compared to the rest of the population, and a parallel‐reduced uptake of health promotion interventions, including a reduced uptake of childhood vaccines, and reduced baby clinic attendances (UNICEF, 2011). However, 68% of the mothers had at least secondary education; a factor that has been previously noted to be better associated with adequate health‐seeking behaviors and uptake of health promotion messages (Makoka, 2013).

In this analysis, there was a statistically significant association between the ages of children who had recent episodes of diarrhea, with the mean age of those who had an episode at 22 months while the mean age of those who had not reported a recent episode at 30 months, *p* = .001. This remained significant in multivariate analysis. In a methodologically similar cross‐sectional study in an informal settlement in South Africa, the highest prevalence of diarrhea was among younger children, less than 12 months of age (Nguyen et al., 2021). In their study, Gupta and colleagues noted 57.69% of diarrhea cases in children aged 7–12 months, and 25.71% in those aged 13–24 months, and a decreasing prevalence with increasing age (Gupta et al., 2015). Not just in Zimbabwe, but globally, the evidence supports a higher occurrence of diarrhea in the younger groups, and therefore a greater need to protect this vulnerable population.

We noted the incidence of a recent episode of diarrhea to be statistically significantly affected by gender, with 16% among females and 19% among their male counterparts, with *p* = .013.

Similar to what has been widely reported in the literature, in this study, we noted decreasing incidences of episodes of diarrhea with increasing maternal education level and increasing wealth quintile. Higher maternal education levels and wealth quintiles have been largely noted to be with higher uptake of health promotion practices, health‐seeking behaviors and hygienic practices, such as frequent hand washing and ensuring access to safe and clean water, particularly in the provinces with lower access to safe toilets and clean water. These factors have been noted to be protective of diarrhea in previous cross‐sectional and case–control studies (Asfaha et al., 2018; Tambe et al., 2015). The mother's level of education was noted in this present study to be also associated with seeking medical attention for a recent episode of diarrhea in the child and corroborates the improved health‐seeking behavior by these mothers. However, contrary to frequent epidemiological findings, we did not note significant associations between episodes of diarrhea and religion, despite the sample representativeness and reduced risk of selection bias. Previously studies have reported the apostolic religious group lacked adequate knowledge on health and disease risk factors.

We also found an association between wealth quintile and chances of seeking medical attention for the recent episode of diarrhea. Individuals with higher wealth are more likely to have higher access to improved water sources and be better educated and therefore more knowledgeable about how best to prevent diarrhea and have more access to improved toilet facilities.

The results are useful in informing pediatric public health policies and strategies for them to be successful in significantly reducing the incidence, morbidity, mortality and significant healthcare costs and burden to society associated with caring for children with diarrheal illnesses not only in Zimbabwe but similar countries in sub‐Saharan Africa.

### 4.1 Limitations

This study provides insights into some of the factors associated with diarrheal disease in children. Its findings are in line with previous literature, and they corroborate what is already known on the topic and needs to be considered in public health policy, strategy and practice. However, the cross‐sectional design sampling technique, and resultant distribution of demographic characteristics, may have prevented all relevant factors associated with diarrheal diseases in children under‐fives to be identified. Cross‐sectional studies do not show a temporal relationship between exposures and outcomes in the same way longitudinal studies would do. In addition, it may also have been underpowered to detect differences in some of the variables, such as the consumption of water from unimproved sources and access to adequate toilet facilities. Therefore, larger studies with a bigger sample size to accord higher statistical power may be required to fully explore this issue.

## 5 CONCLUSION

Diarrhea among children under‐five remains a significant public health challenge in Zimbabwe. Continuous efforts to reduce the incidence of Diarrhea are required and rest upon adequately understanding and addressing risk and protective factors and responding adequately to this public health challenge. Continued monitoring is therefore required to inform pediatric public health policies and strategies for them to be successful in significantly reducing the incidence, morbidity, mortality and significant healthcare costs and burden to society associated with caring for children with diarrheal illnesses.

## CONFLICT OF INTEREST

The authors declare that they do not have any conflict of interest.

## ETHICAL APPROVAL

Procedures and questionnaires for standard Demographic Health Surveys (DHS) have been reviewed and approved by the ICF International Institutional Review Board (IRB). Additionally, country‐specific DHS survey protocols are reviewed by the ICF IRB and typically by an IRB in the host country specifically the Medical Research Council of Zimbabwe. Written informed consent was obtained from all ZDHS participants.

## Data Availability

The data that support the findings of this study are available from the Demographic and Health Surveys (http://www.measuredhs.com) upon reasonable request.
